# Respect, interaction, immediacy and the role community plays in registering an organ donation decision

**DOI:** 10.1371/journal.pone.0263096

**Published:** 2022-01-26

**Authors:** Gail Moloney, Michael Sutherland, Leah Upcroft, Rachel Clark, Parul Punjabi-Jagdish, Suzanne Rienks, Alison Bowling, Iain Walker

**Affiliations:** 1 Faculty of Health, Discipline of Psychology, Southern Cross University, Coffs Harbour, NSW, Australia; 2 Intensive Care Unit, Mid North Coast Local Health District, NSW, Australia; 3 NSW Organ and Tissue Donation Service, South Eastern Sydney Local Health District, NSW Australia; 4 Faculty of Health, Discipline of Psychology, Southern Cross University, Coffs Harbour, NSW, Australia; 5 Research School of Psychology, College of Health and Medicine, Australian National University, Canberra, Australia; Illawarra Shoalhaven Local Health District, AUSTRALIA

## Abstract

**Background:**

Registering a donation decision is fundamental to increasing the number of people who donate the organs and tissues essential for transplantation, but the number of registered organ donors is insufficient to meet this demand. Most people in Australia support organ donation, but only a third have registered their decision on the Australian Organ Donor Register (AODR). We addressed this paradox by investigating how feelings of community, engendered through an ethic of hospitality and care and a non-proselytizing dialogue about organ donation, facilitated the decision to register.

**Methods:**

An Immediate Registration Opportunity was set up in a large public hospital in NSW, Australia. The public was approached and invited to engage in an open, respectful dialogic interaction that met people where their beliefs were and allowed their concerns and fears about donation to be discussed. This included a survey that measured positive and negative beliefs about organ donation, mood, atmosphere, and feelings of community coupled with an on-the-spot opportunity to register their donation decision.

**Results:**

Over four days, we interacted with 357 participants; 75.5% (210) of eligible-to-register participants registered on the AODR. Generalized Structural Equation Modelling highlighted that as connection to community increased, so did the salience of positive beliefs about organ donation. Positive beliefs, in turn, were negatively correlated with negative beliefs about donation and, as the strength of negative beliefs decreased, the probability of registration on the AODR increased. Participants who registered on the AODR reported stronger connection to the broader community than participants who did not register.

**Conclusion:**

A respectful non-judgmental interaction that allows beliefs and concerns about organ donation to be discussed, coupled with an immediate opportunity to register, encouraged registration. Within this framework, feelings of belonging to a community were a key determinant that enabled many to make the decision to register.

## Introduction

Few people disagree that organ donation saves lives, helps others and benefits society [1–3]. However, many people add a *but* to this statement [4]. *But*, will I really be dead if I agree to be a donor? *But* I don’t want to be cut up. *But* I won’t be whole if I donate. *But* what about my eyes? *But* I am not sure what my religion thinks [4,5]. One reason why marketing and community awareness programs do not explicitly address these *buts* may be a fear that doing so will deter people from considering organ donation [6,7]. We investigated whether this was the case by inviting the public to engage in a respectful, non-proselytizing *open* dialogue about organ donation coupled with an immediate on-the-spot opportunity to register their donation decision.

### Australia’s donation system

Australia has an opt-in donation system, where the public is actively encouraged to register their donation decision on the Australian Organ Donor Register (AODR). The DonateLife website allows people to register a “Yes to donate all organs and tissues” whereas the Australian Government Services website (MyGov) and the hard copy AODR registration form, offer a choice in how people can register: a "Yes" to donate all organs and tissue, or a “Yes” to specific organs and tissue, or a “No" to donation [8]. Registration is important because it facilitates timely national access to a person’s donation decision for medical personnel, who will then discuss the decision with the next-of-kin and other family members. In 90–93% of cases, the next-of-kin will honour a consent decision if it was registered on the AODR. This figure drops to 44–51% when the decision is unknown [9,10], primarily because the next-of-kin have to make the donation decision themselves [11]. This can add further stress to a time of grief, and “lead to the decision of the least discomfort, a donation decline” [11, p.647]. Only 34% of people in Australia have registered their donation decision, and 39% in NSW [12], the location of this study.

In Australia, anonymity in cadaveric donation is a social, ethical and legal norm [13]. Donation is facilitated by healthcare professionals with no direct contact between the donor’s family and the recipient [14]. Nevertheless, there are communities, families, and individuals who want to lift this veil of anonymity and know the identity of the donor or recipient. This suggests these families or individuals may feel donating their loved one’s organs creates a communal tie or a relationship with the recipient. Similarly, registering a donation decision means thinking about organ donation in relation to someone but no-one-in-particular–an abstract, hypothetical other. For some people, this may be at odds with their connection to a cultural, religious, or social community [15].

### Community

Feelings of belonging to a community have been defined as the extent to which a person perceives they are connected to community, through actual ties or a psychological attachment [16]. Community-belonging has been shown to motivate human action, facilitate positive emotions and counter the biological stress of isolation [17,18]. At an individual-level of analysis, a connection to community has been associated with positive health-behaviour change in relation to exercise, weight loss and diet, through exposure to health-related behavioural norms or attitudes [16]. Similarly, community-belonging has been related to blood donation through civic reciprocity, which is also a strategy used to promote organ donation. Blood donation is seen as a way to give back, with the strength of civic reciprocity significantly influencing long-term blood donation [19]. At a group or positional level of analysis [20], belonging has been linked to strength of identification with social groups arising through intra-group similarity and perceived protypicality [21]. Caideras [22] found that engagement and dialogue on social media about organ donation increased when a community was established before information was disseminated. At a macro-social level of analysis, community is a socially-constructed space in which people enact representations through shared identities [23]. Identity is grounded in community through what is perceived to be shared or not shared with others [24]. Campbell and Jovchelovitch [25] speak of community in relation to a group of people who share identity, socially-constructed understandings, conditions of access and constraint to resources and is experienced when all three aspects are enacted. The link between perceptions of community-belonging, representations, and the organ donation decision has yet to be investigated.

### The decision to register

The decision to register a donation decision is inextricably linked to what people understand about organ donation, which is typically acquired through societal discourse, including the media, and the country’s institutional approach to organ donation [4]. Conceptualized within social representations theory [26], organ donation is more than an individual’s attitude or decision. It is a shared understanding constructed and shaped by the exchange and interaction processes within a group or society. As symbolic collectively-concerted patterns of thinking, social representations give meaning to unfamiliar, new, or threatening social issues, particularly those that emanate from the medical and scientific realms [26,27]. Representations are social because they are created through communication with others [27], and because they are shared with others. They provide the parameters for discussion, argument, and negotiation about a social issue [4]. This does not exclude or negate personal experience or thought; rather, it acknowledges that how a person understands or what is associated with a social issue has its origins in mutual interaction [4].

Defined as “values, ideas and practices” [28, p8] representations are systems through which a society or a group come to understand social issues. They may be heterogeneous, contradictory, and ambiguous with aspects becoming more or less salient depending on the context in which the representation is elicited. This has been seen in natural disasters involving whole communities [29–31], such as the dramatic and unprecedented increase in blood donations in response to the 2009 Victorian bush fires in Australia [31]. Explained theoretically, the context of the devastation, injury, and death caused by the bushfires made the belief that donating blood could help others particularly salient, overshadowing the fear and pain typically associated with needles and donation [30].

In Australia, organ donation is promoted as an altruistic *gift* that saves and transforms lives [1,8,32]. However, promoting the benefits of donation as an altruistic gift is an asymmetrical account of what donation involves, because it fails to acknowledge the heterogeneous nature of what is socially understood about donation [5,32,33]. For many people, thinking about organ donation, or the process of registering an organ donation decision, confronts socially-validated boundaries around life and death, bringing to the fore unspoken concerns and fears associated with the donation process. *Will I really be dead if I agree to be a donor*? *Will the doctors try as hard to save me if I am a donor*? *Will I be disfigured*? These concerns and fears do not negate the altruistic sentiments associated with donation as a gift of life; rather, they co-exist and are often the reason why many people will avoid or delay registering a donation decision even when they favour donation [4,34,35]. It is the relationship between positive altruistic beliefs and the negative concerns and fears, rather than the strength of each belief, that predicts the likelihood that someone will register a donation decision or sign a donor card [5,33,36,37].

### Social context and registration

The context in which beliefs about organ donation are elicited can also influence registration behaviour. Rodrigue and colleagues [38] report that likelihood and intention to register were greater when participants watched videos that elicited positive emotions rather than videos that did not elicit these emotions. Similarly, Moloney and colleagues [11] found as perceptions of a shared positive, upbeat mood increased at the Grand Final of a sporting event, so too did the salience of positive beliefs associated with organ donation. In turn, negative beliefs decreased and the likelihood of registration increased. Conversely, environments associated with negative affect, such as the Department of Motor Vehicles in the US, have been linked with increased negative emotions [39], which were subsequently associated with a decreased intention to register [40].

Theoretically, the context elicits different aspects of the heterogenous representational field. Core beliefs interface with the context through peripheral beliefs, which are fluid and manifest in discourse according to the context [11,32,41]. For example, life and death were central to how organ donation was understood in our early Australian studies [4,41]. These central, or core, beliefs manifested in discourse as a gift of life *and* the mechanistic removal of body parts, but the context, or the questions asked, influenced which of these aspects were elicited. In the current study, we were interested whether creating an ethic of hospitality and care would create a context of community, which, in turn, would strengthen the salience of positive beliefs that people associated with donation, thereby decreasing the salience of negative beliefs.

### Current study

The study was conducted in the foyer of a large busy public hospital in New South Wales (NSW), one of the most culturally diverse and populous states in Australia [42]. Although organ donation has a natural synergy with the medical environment [33], many people find medical environments alienating, "jarringly unfamiliar", and unnatural spaces [43, p2]. Considering organ donation and, therefore, one’s mortality may sharpen this sense of unease. Chan [44] highlights the centrality that a care ethics approach has in all phases of the donation process in particular, the need to provide people with an opportunity to engage with the idea of organ donation before a donation request is a reality. This approach may also evoke a sense of community through the co-construction of a shared understanding of organ donation. Proselytizing, or urging people to take a predefined action, does not leave space for the construction of this shared understanding. Therefore, the aim of this study was to investigate how registration behaviour relates to community-belonging and beliefs associated with organ donation, when the public are invited to engage in a non-proselytizing, respectful interaction about organ donation with an opportunity to register their donation decision on the Australian Organ Donor Register.

In a dialogical encounter, an individual is not an isolated solipsistic self [45] but an inherent part of the interaction [46]. Preceded by phatic communication that signals an intention to establish an interactive relationship, a dialogical interaction about organ and tissue donation is about fostering an understanding of what the other thinks. Embedded in care and openness, the interaction is orientated to the other individual. Rather than being an asymmetrical proselytizing dialogue about the benefits of organ donation, the dialogical interaction acknowledges the individual as a co-creator of the exchange, enabling co-created understandings which evoke a sense of community. Beliefs and views about donation are acknowledged *before* an alternative fact-based statement is offered [47], creating the potential for shared social knowledge and social norms to be brought together [45] as an antecedent to compatibility, goodwill, and acceptance [48].

## Materials and method

### Sample

Three hundred and fifty-seven participants (*M*_age_ = 45.9, *SD* = 17.1) interacted with the Immediate Registration Opportunity over four days (Table 1).

**Table 1:** Participant demographics and registration.

		Total *(N/n)*	%
**Total number of interactions**		357	100
**Previously registered**		79	22.1
**Eligible and not previously registered**		278	77.9
**Did they register at the stall?**	Yes	210*	75.5
	No	31	11.2
	Would like more information	33	11.9
	Took forms home	4	1.4
	Took a registration card	95	45.2
**Gender**	Female	225	63.0
	Male	91	25.5
	Non-binary	1	0.3
**Nationality/Cultural identification**	Australian	243	68.1
	British	10	2.8
	Chinese	10	2.8
	Nepalese	6	1.7
	Indian	5	1.4
	Indigenous Australian	5	1.4
	Irish	5	1.4
	Singaporean	4	1.1
	(*n* = <4)	44	12.4
**Religious or spiritual group**	Christian	99	27.2
	Atheist/Agnostic/Secular	15	4.2
	Hindu	9	2.5
	Judaism	6	1.7
	Islamic	3	8
	(*n* = <4)	28	7.9
**Know a donor/ recipient**		191	53.5
**Aware drivers licence phased out**		162	45.5

* All participants who registered, registered a decision to donate all or some organs and tissue. Missing data (no response given) for gender (11.5%), cultural/national identification (48%), and religious/spiritual group (55.2%).

**57.7% of females registered from those who identified as female and 51.6% of males registered from those who identified as male. Not all participants identified a gender.

### Location

The Immediate Registration Opportunity was located in the foyer adjacent to the main entrance of a public hospital in New South Wales, Australia during "DonateLife Week" (July 2019). DonateLife week is a national awareness week in Australia dedicated to promoting organ and tissue donation [49]. The registration opportunity was affiliated with the University and DonateLife. The five members of the research team were aged between 25 and 62, male and female, and from different social, cultural and religious backgrounds, including an organ donation doctor with specific expertise in donation and transplantation, and a team member able to converse in six different languages. All members of the research team were trained to offer the Immediate Registration Opportunity, had knowledge about organ donation, and were skilled in how to engage in a non-proselytising dialogue, and respond appropriately to questions.

#### The immediate registration opportunity

The Immediate Registration Opportunity offers members of the public an immediate on-the-spot opportunity to register their donation decision on the Australian Organ Donor Register. The DonateLife stall is set up at the location and the public are approached and invited to engage in conversation about organ donation and complete a short survey. During this interaction, participants are encouraged to ask questions, comment and raise concerns about organ donation and the registration process. Participants are asked at the end of the survey (and in person) if they would like to sign the Australian Organ Donor Register now. They are also made aware that they have a choice in how they register, and their decision can be changed at any time. If they reply yes, they are given an Australian Organ Donor Registration form to complete on-the-spot. All participants are invited to take DonateLife merchandise (stickers, hats, cups). Participants who register are also invited to take an *I have registered on the Australian Organ Donor Register* card (a custom-designed fridge magnet) to give, to or take a photo of and send via social media or email. to their family, friends, and work colleagues.

### Materials

#### The survey

The 25 item self-report survey measured participants’ beliefs about organ donation, registration status with an option to register, mood, atmosphere and feelings of community and belonging at that moment, and demographic information. The survey was developed by Moloney and colleagues. Organ donation beliefs were conceptualised within social representation theory [4,41], as multidimensional scales [36,37]. The current survey also included questions about mood and atmosphere adapted from previous research [11] and feelings of community and belonging [see 24,25].

*Beliefs about organ donation*. Beliefs were measured using a previously validated 12 7-point Likert scale with items ranging from 1 *= strongly disagree* to 7 = *strongly agree* [11,32,33]. The 12 items have been reported as a four-factor [11,32,33] solution [see also 50]. Three items measured Gift of Life beliefs (α = .73; e.g. *The act of donation is about giving life to someone else)*; three items measured Benefits of Donation to the Self (α = .77; e.g. *Donating a body part would enable that part of a person to live on);* three items measured Negative Consequences associated with Donation (α = .74; e.g. *The thought of a body being cut up or taken apart after a person has gone makes one feel uneasy*); and three items measured Concerns over the Medical Care associated with organ donation (α = .78; e.g. *It is hard to trust the doctors involved in organ donation)*.

At the end of the first page, and in person, participants were asked, *"Were you aware that indicating your organ donation wishes on your Driver’s Licence has been phased out*? *(Yes/ No) "* followed by "*Would you like to sign the Australian Organ Donor Register now*?" ("*yes*, "*no*", "*I have already registered*" or "*I would like more information")*.

Mood was measured by "*Please list the five words that best describe your mood now*".

Atmosphere was measured by "*Please list the five words that best describe the atmosphere here*".

Community was measured on four 7-point Likert scale items from 1 *= not at all* to 7 = *a lot*. Participants were asked: "*At this moment*, *to what extent do you*: *feel part of a community*? *Feel you share similar feelings/mood to the other people here*? *Feel a communal sense of positivity*? *Feel that making this decision connects you to a broader community*?*"*.

Participants were asked their age, gender, nationality/culture, religious/spiritual group (if any), and whether they knew anyone who had donated or received an organ.

The study was approved by the University Human Research Ethics Committee and the Chair of the Hospital Human Research Ethics Committee. Participants were given written and verbal information about the study, and the completion of the self-report questionnaire indicated informed consent (ECN-18-138).

### Procedure

#### Enacting an ethic of hospitality

Each encounter began with a warm greeting and a general statement indicating a desire to have a conversation on the person’s views about organ donation and transplantation. Emphasis was placed on the person, acknowledging their views and acknowledging where that person was at, before offering an alternative fact-based statement, if and when appropriate (e.g. ’yes indeed, the vast majority of Australians do hold that belief but did you know…’). Ensuing options for discursive chat or humorous asides were always taken to ensure the interaction was enjoyable. The person was invited to complete a brief survey on their beliefs about donation, with someone on hand to answer any questions, acknowledge and discuss any concerns. Emphasis was placed on the validity of the participant’s answers, stressing there were no correct or incorrect answers. An invitation was given for the participant to register their donation decision on the Australian Organ Donor Register (using a hard copy form) ensuring the participant knew they had a choice in how they registered and that their decision could be changed at any time. In a sense, this approach encouraged people to register their donation decision by not specifically encouraging that as an outcome. It was about a moment in time, a person-to-person interaction, and an exchange of views. The natural segue to this was often an on-the-spot registration.

## Results and discussion

### Results

In total 357 participants (*M*_age_ = 45.92; *SD* = 17.09) interacted with us over the four days. Two hundred and seventy-eight participants (77.9%) had not previously registered despite being eligible to do so, and 79 participants (22.1%) were already registered on the AODR (Table 1). Two hundred and ten (210) participants (75.5% of those eligible to register) registered on one of the four days the Immediate Registration Opportunity was offered.

It was not possible to collect data on participants’ reasons for visiting the hospital or their occupations. The survey was short due to the constraints of conducting the research in the entrance foyer of a busy public hospital. However, we were aware from our conversations, that participants were associated with a wide range of occupations including medical and allied health staff, administrative staff, café workers, florists, cleaners, patients, people attending specialist clinic appointments, as well as friends and families of patients.

#### Beliefs about donation

A Confirmatory Factor Analysis using maximum likelihood estimation procedures was performed on all responses to the 12 items measuring beliefs about organ donation. A four-factor model offered the most parsimonious fit for the data (Tables 2 & 3).

**Table 2:** Confirmatory factor analysis details of fit.

df	*X* ^*2*^	*X*^*2*^/df	CFI	TLI	RMSEA
48	72.39	1.65	0.979	0.967	.043

CFI: Comparative fit index, TLI: Tucker-Lewis index, RMSEA: Root mean square error of approximation.

**Table 3:** Standardized factor loadings for 12 items of the 4-factor model.

	Factor			
Items	Gift of Life	Benefits to Self	Medical Care	Negative Cons
Donating organs at death is a way of putting some parts of the body to beneficial use	.80			
The act of donation is about giving life to someone else	.88			
Organ donation is about helping other people	.76			
Becoming an organ donor makes a person feel proud		.64		
Deciding to donate one’s organs at death adds extra meaning to life		.80		
Donating a body part would enable that part of a person to live on		.64		
By agreeing to be an organ donor, doctors might declare a person dead too soon			.70	
It is hard to trust the doctors involved in organ donation			.75	
Doctors won’t try as hard to save the life of someone who is a potential donor			.79	
A person who is considering donating organs will feel like they have a piece missing when they are buried or cremated				.77
The thought of a body being cut up or taken apart after a person has gone makes one feel uneasy				.79
Organ donation leaves the body disfigured				.66

Overall, participants strongly agreed with the Gift of Life and Benefits to Self beliefs. Scores for Negative Consequences associated with donation and Concerns about Medical Care were relatively low (see Table 4).

**Table 4:** Means (*SD*), Pearson Product-Moment correlations and Cronbach’s alpha for the Beliefs about Organ Donation sub-scales (*N = 330)*.

	Mean (SD)	1	2	3	α
Gift of Life	6.78 (0.65)				.85
Benefits to Self	6.01 (1.30)	.41**			.72
Negative Cons	2.19 (1.45)	-.29**	-.11*		.78
Medical Care	1.90 (1.40)	-.24**	-.05	.68**	.79

*p < .05.

**p < .01.

Mean scale scores for the four community items were moderately strong and significantly correlated (Table 5).

**Table 5:** Means (*SD*), and Pearson Product-Moment correlations for four community items.

	Mean	*SD*		*1*		*2*		*3*	
At this moment, do you feel:									
You are part of a community	5.75	1.28							
You share similar feelings /mood to the other people	5.62	1.31		.59**					
A communal sense of positivity	5.87	1.27		.59**		.63**			
That making this decision connects you to a broader community	5.80	1.51		.42**		.47**		.60**	

**p < .01.

#### Community, beliefs about organ donation, and registration behavior

**Fig 1:**
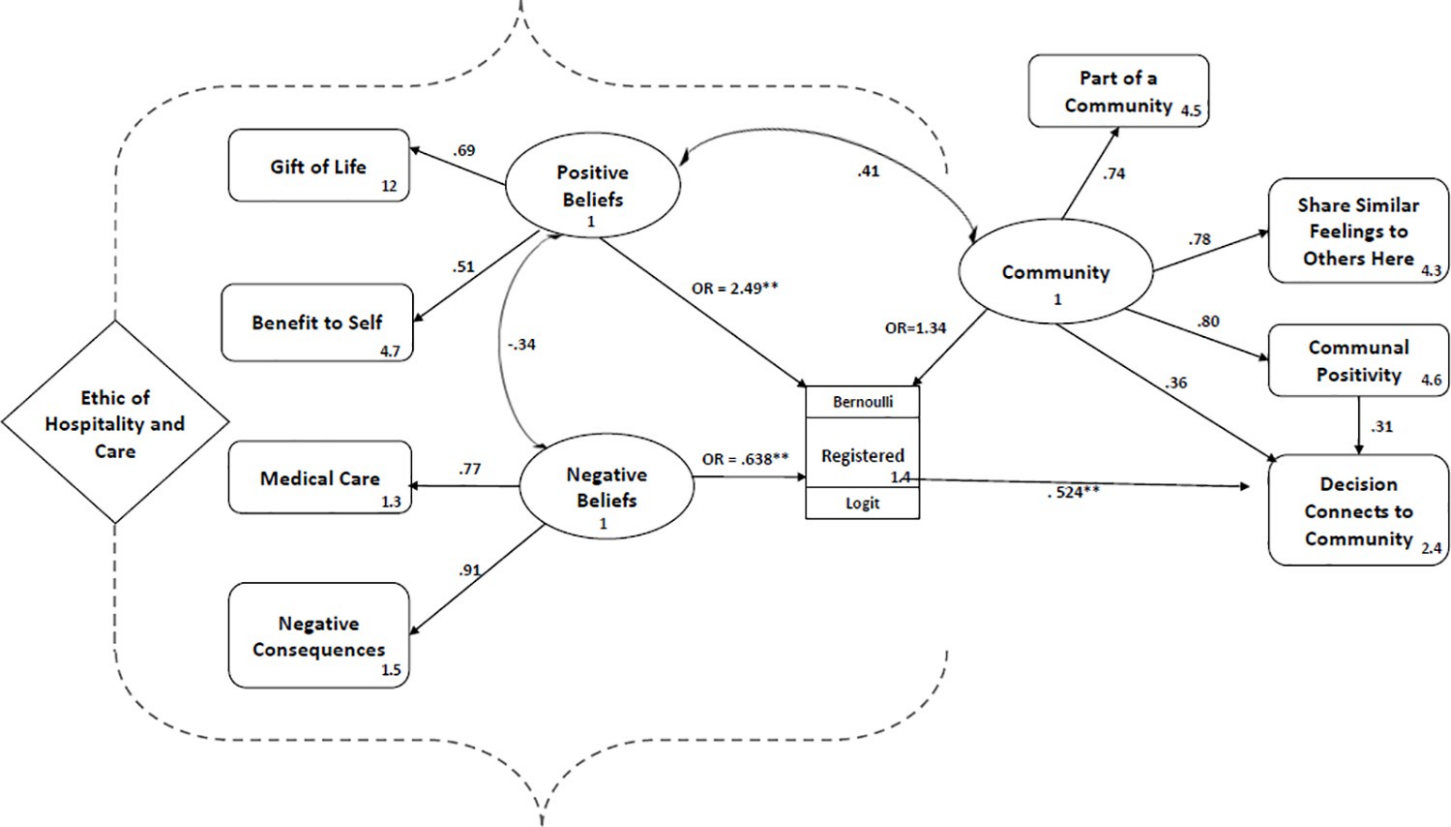
Generalized structural equation model with factor loadings, odds ratios and correlations.

A path analysis using a Generalized Structural Equation Model was used to investigate the relationship between the responses to the community items, beliefs about organ donation, and registration status (registered on the day/ did not register on the day). The four community items were treated as continuous variables, as were Gift of Life, Benefits to Self, Negative Consequences and Concerns over Medical Care. Previous research has demonstrated a relationship between positive and negative beliefs and the probability of registration [5,11,33], and community as a correlate with positive beliefs [18]. The outcome variable, registration status, was a binary variable modelled with a Bernoulli distribution and a logit link function. We also explored whether registration on the day (yes, no) would predict scores on the item, "*Making this decision connects me to a broader community*" (Fig 1).

Community was significantly correlated with Positive Beliefs, which was significantly negatively correlated with Negative Beliefs. Negative Beliefs significantly predicted Registration, with the odds of registering decreasing by 36.2% with each unit increase in Negative Beliefs. Positive Beliefs also significantly predicted Registration, with the odds of registering increasing by 149% with each unit increase in Positive Beliefs. Registration predicted “*Making this decision connects me to a broader community*", with the difference in those who registered being .53 of a unit higher than those who did not register (Fig 1).

Community did not predict registration in the context of the other variables. As Community was significantly correlated with Positive Beliefs, it is likely that these two variables share variance in the prediction of Registration. To assess this, the model was rerun with Positive Beliefs omitted and Community as the only predictor (see Table 6). Community now significantly predicted Registration. A Log Ratio test revealed the two models were significantly different (χ2 = 6.88, p = .009). Consequently, although Community was a significant predictor when entered on its own into the model, it does not uniquely predict registration when Positive Beliefs is included, due to variance shared between these two variables.

**Table 6:** Comparison of fit for generalised structural equation models.

Model	N	-2LL	df	AIC	BIC
Model 1 (with Positive Beliefs)	357	7601.8	31	7663.819	7784.029
Model 2 (without Positive Beliefs B)	357	7608.7	30	7668.704	7785.036

AIC: Akaike’s information criterion BIC: Bayesian Information Criterion.

#### Mood and atmosphere

Previous research has indicated the importance of the context in which discussions about organ donation take place (12, 39, 40,41), particularly perceptions of the mood and atmosphere at that time. To investigate how interacting with us in a hospital foyer was perceived, participants were asked to list the first five words that described their mood and the atmosphere at that moment. The elicitations were homogenized into word categories using root words and closely related synonyms and then grouped into broader categories (Table 7). A total of 445 first words were elicited for atmosphere. The most frequently elicited categories suggested that overall the atmosphere was perceived to be very positive (e.g. good, happy, calm, friendly, helpful, supportive, great, welcoming). Similarly, a total of 530 words were elicited for mood. The most frequently elicited categories suggested that overall participant mood was very positive (e.g. happy, good, relaxed, content, fine, thoughtful).

**Table 7:** Categorized elicitations with percentage response.

Atmosphere	Category	%	Homogenized words in category
	Positive	67.80	Good, positive, happy, calm, friendly, helpful, supportive, great, welcoming, encouraging, enthusiastic, relaxed, warm informative, comfortable, peaceful, fine, open
	Busy	13.03	
	Cold/Negative	2.45	
	Neutral/normal	2.25	
	Noisy	1.77	
	Professional	1.57	
	Hospital-like	1.33	
	Other	9.80	
Mood	Positive	76.00	happy, good, relaxed, positive; content, fine, thoughtful, ok, helpful, excited, grateful, hopeful, pleased, and optimistic, encouraged, community- minded
	Stressed/anxious	4.71	
	Tired	4.15	
	Busy	3.77	
	Energetic	.18	
	Other	11.19	

### Discussion

We investigated the role played by a connection to community when people were asked to consider their beliefs about organ donation and offered an opportunity to register their donation decision on the Australian Organ Donor Register.

The results showed that participants who reported stronger Positive Beliefs about donation also reported stronger feelings of belonging and being part of a community. Moreover, as reports of Positive Beliefs strengthened, the Negative Beliefs associated with donation decreased, and, as Negative Beliefs about donation decreased, the likelihood of registration increased. It is plausible that our interactions, encapsulated within an ethic of hospitality and care, fostered the connection to community that many participants reported, but we cannot evidence this. But, as a result of offering the Immediate Registration Opportunity, 75% of people who had not yet registered, registered with us on one of the four days the registration opportunity was offered. This figure is double the current national registration rate (34% NSW state: 40%) [12]. Moreover, responses to the item "*Making this decision connects you to a broader community*" were significantly higher for those who did register compared to those who chose not to register.

An alternative explanation for the strong community-belonging scores is self-selection. Participants who already held strong feelings of community self-selected to engage with us about organ donation and registration. Irrespective of whether this was the case, the findings do highlight a link between feelings of community and registration behaviour.

#### Community-belonging within an ethic of hospitality and care

Easterbrook and Vignoles [21] found that perceptions of similarity with others in the group, along with prototypicality, were related to feelings of belonging. Participants in this study reported that “at that moment” they shared similar feelings to other people, suggesting they identified with the other people at the Immediate Registration Opportunity–other participants, the staff or both. The participants also reported they felt a communal sense of positivity, and being part of a community.

Howarth [24], and Campbell and Jovchelovitch [25] also identify the importance of a shared identity in relation to feelings of belonging, but suggest that shared identity emerges within shared representations. When we approached people, we opened the interaction by emphasising that this would be a non-proselytizing, respectful dialogue about organ donation. Theoretically, this is through negotiating an inter-subjective understanding of the other person’s beliefs [23], which Wagner [48] describes as boot-strapping. Becoming conscious of our own knowledge or representational systems as well as those of the other person and positioning ourselves as learners so as to understand the beliefs of the other person from their social and culturally embedded perspective [48].

The rich cultural and religious heritage of Australia’s multicultural society includes 300 different ancestral groups, 100 different religions and 300 spoken languages [42]. Organ donation beliefs are often embedded in different understandings of health and well-being, and rarely a linear product of religion or ethnicity alone [5]. It is plausible that participants felt the other participants and staff at the Immediate Registration Opportunity held similar beliefs about organ donation and similar questions; otherwise why would they be there talking with the staff. Most participants spent between five and fifteen minutes interacting with us. There were often several participants at the stall at any one time talking, completing the survey or taking photos of the registration cards. The atmosphere was positive and welcoming, and the mood happy and relaxed–descriptors at odds with how medical environments are typically perceived [43]. While we cannot evidence whether our interactions, encapsulated within an ethic of hospitality and care, fostered the community-belonging responses, the salience of positive beliefs about donation was positively associated with the strength of community-belonging.

These findings highlight how pivotal it is that people are welcomed and invited to engage in a *respectful*, *open* dialogue about organ donation. This is important as research in the US suggests that interventions designed to increase registration are more likely to be successful in contexts where people experience positive rather than negative affect [38,40,51]. Similarly, changing the "culture" at the Department of Motor Vehicles in the US through donor-centric interventions (with a focus on customer experience, employee education, and office decorations) was shown to increase the number of people who registered at these locations [52].

#### Conversations about organ donation and the registration decision

From our observations, it was apparent that physically approaching people was crucial to initiating an interaction. Equally important was the need to signal early that this engagement would be open and oriented to the person rather than a proselytizing dialogue about organ donation. Hence, a welcoming smile, a simple warm gesture, and a non-obtrusive move towards the person initiated our interactions. We noted that when we did not actively approach people, they seldom stopped, casting a curious glance instead before continuing on. These findings concur with research that found registration rates at "unstaffed" immediate registration kiosks were significantly less than at staffed stalls [53,54]. A hallmark of our interaction was listening to the participants, acknowledging their beliefs, and, then, when appropriate, respectfully addressing any concerns they had.

The location of our Immediate Registration Opportunity, in the foyer of a large public hospital in a major city in Australia, allowed us to engage with people from diverse social, cultural, and religious backgrounds. Understanding how the normative sentiments associated with organ donation—the gift of life, saving lives, helping people; can inadvertently silence the concerns and fears that people hold about donation is crucial in navigating these conversations. People may feel hesitant or even embarrassed to ask questions, raise concerns or fears in light of the strong institutional messaging around organ donation as a “Gift” that saves lives [1]. Ensuring there is the space and respect to do so is crucial. Inviting participants to complete the Beliefs about Organ Donation survey (which asked about commonly held concerns and fears in addition to positive beliefs) also acknowledged the concerns participants held and provided them with permission to ask for further information.

#### Community as a context

The context in which the Immediate Registration Opportunity is offered can affect the salience of the beliefs about organ donation and subsequently registration behaviour [11]. In this study, the context of a connection to community was positively correlated with strong positive beliefs about donation. Theoretically this reiterates how aspects of the representational field associated with organ donation become more or less salient depending on the context in which the representation is elicited [4,41].

Experiencing a connection to others and to community may highlight the positive beliefs associated with organ donation, which, in turn, decrease the salience of negative beliefs. This has implications for the Intensive Care Unit, where the converse may occur if family members are asked to consider for the first time the donation decision of their loved one–when they are feeling overwhelmed, stressed and grief-stricken [55].

### Limitations and future research

Conclusions drawn from field research must be more cautious than research conducted in the laboratory or with an experimental survey design. A limitation of the present research is the lack of a control group–a "no-staff immediate registration condition"—to evidence the effect of respect and interaction on immediate registration rates, and to further tease out the role of community. However, research by Salim [53] and Siegal [54] has demonstrated that unstaffed registration kiosks are less effective than those that are staffed. Moreover, the notable disparity between the registration rates over the four days we offered the initiative (75%) compared with the registration rates for NSW (40%) and nationally (34%) [12] speaks to the likelihood that a sense of community does not, on its own, lead to people to register. A further limitation was the gender skew of participants towards females. Although more females than males interacted with us over the four days, further analyses revealed a similar percentage of females and males registered (from those who identified as female and male) (see Table 1).

We suggest that a connection to community is important to consider further, particularly the relationship this has with the principles of respect, interaction and immediacy that underlie the success of our Immediate Registration Opportunity [5,11,33]. Respect for the diversity of beliefs held about organ donation, both positive and negative. Non-proselytizing interactions so all people can consider their beliefs about organ donation, ask questions, and raise concerns and fears without judgement. And, an immediate on-the-spot opportunity to register a donation decision, whatever that decision may be.

It is also important to consider research [56,57] that has found self-efficacy and vested interest are important in increasing registration rates in the Department of Motor Vehicles in the US. Fostering community belonging in this context, which has been associated with negative affect [39], may further increase the number of people who register. This could be important to consider in countering some of the barriers to the opt-out donation system in countries such as the UK [see 57].

Importantly, this approach has the potential to ripple and engage others in the donation conversation. Forty-five percent of participants who registered with us shared custom-designed registration cards with family and friends–sending a photo of the card, giving it to someone, or putting the card on the fridge, desk or in the office kitchen, thereby creating a stimulus for further conversations with family, friends, and work colleagues about organ donation and registration.

## Conclusion

Registering a donation decision is fundamental to increasing the number of people who donate their organs and tissues, and, therefore, fundamental to enabling transplantation for thousands of people who would otherwise die, spend years on dialysis or live a life in pain. Most people support the idea of donation, but only a third of the eligible population in Australia have registered. The challenge for Australia and for many other countries is *how* to increase registrations so that *everyone* registers their donation decision.

The task now is to scale these social mechanisms and implement them in the broader community. To-date, the research findings have been incorporated into awareness and engagement activities undertaken by DonateLife, community partners and volunteers. We envisage the scaling of these mechanisms, which is currently in progress, will add to this body of work and be relevant for all Australian and international donation agencies. Understanding the principles that facilitate the decision to register is the first step to mobilizing a community of shared understanding, a new wave of registration, and an increase in donation and transplantation towards levels consistent with the needs of the population.

## Supporting information

S1 File (PDF)
