# Supplementary material for: Respect, interaction, immediacy and the role community plays in registering an organ donation decision
Source: PLoS One. 2022 Jan 26;17(1):e0263096. doi: 10.1371/journal.pone.0263096 (PMC8791491; doi:10.1371/journal.pone.0263096)
Supplement: S1 File — (PDF) [file pone.0263096.s001.pdf]

**How much do you agree/disagree with these statements?**	Strongly disagree						Strongly agree
1. The act of donation is about giving life to someone else	1	2	3	4	5	6	7
2. Donating organs at death is a way of putting some parts of the body to beneficial use	1	2	3	4	5	6	7
3. Organ donation leaves the body disfigured	1	2	3	4	5	6	7
4. By agreeing to be an organ donor, doctors might declare a person dead too soon	1	2	3	4	5	6	7
5. The thought of a body being cut up or taken apart after a person has gone makes one feel uneasy	1	2	3	4	5	6	7
6. A person who is considering donating organs will feel like they have a piece missing when they are buried or cremated	1	2	3	4	5	6	7
7. Organ donation is about helping other people	1	2	3	4	5	6	7
8. Donating a body part would enable that part of a person to live on	1	2	3	4	5	6	7
9. Deciding to donate one's organs at death adds extra meaning to life	1	2	3	4	5	6	7
10. It is hard to trust the doctors involved in organ donation	1	2	3	4	5	6	7
11. Becoming an organ donor makes a person feel proud	1	2	3	4	5	6	7
12. Doctors won't try as hard to save the life of someone who is a potential donor	1	2	3	4	5	6	7

13. What are your thoughts after reading these statements?

14. Were you aware that that indicating your organ donor decision on your driver's license has been phased out? ☐ Yes ☐ No

15. **Would you like to register your donation decision on the Australian Organ Donor Register now?**

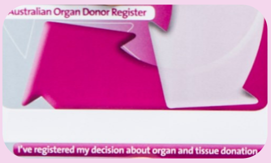

☐ Yes ☐ No

☐ I have already signed ☐ I would like more information

16. We are interested in how you perceive the context you are in at this moment; that is, how *you* would describe your mood and the atmosphere *now*. Please list the first 5 words that spring to mind:

Your mood now ______________________ The atmosphere here ______________________

______________________ ______________________

______________________ ______________________

______________________ ______________________

______________________ ______________________

**At this moment, to what extent do you:**	*Not at all*						*A lot*
17. Feel part of a community	1	2	3	4	5	6	7
18. Feel you share similar feelings/mood to the other people here	1	2	3	4	5	6	7
19. Feel a communal sense of positivity	1	2	3	4	5	6	7
20. Feel that making this decision connects you to a broader community	1	2	3	4	5	6	7

Can you please tell us:

21. Your age _________

22. Your gender ☐ Male ☐ Female ☐ Non– binary I would like to specify _____________

23. Your Nationality _________________ Cultural group__________________

24. Religious/ spiritual group _______________

25. Do you know anyone who has donated an organ or received an organ transplant? ☐ Yes ☐ No

Would you like to comment: ____
